# Supplementary material for: Identification of differentially expressed genes and proteins related to diapause in Lymantria dispar: Insights for the mechanism of diapause from transcriptome and proteome analyses
Source: PLoS One. 2025 Jun 25;20(6):e0316065. doi: 10.1371/journal.pone.0316065 (PMC12192293; doi:10.1371/journal.pone.0316065)
Supplement: S1 File — (DOCX) [file pone.0316065.s001.docx]

Supplementary Tab.1 Sequencing Data Quality Assessment and Mapping Statistics

| Sample Name | Clean reads (Gb) | Q30 (%) | GC content (%) | Mapped ratio (%) |
| --- | --- | --- | --- | --- |
| LN1 | 8.03 | 93.75 | 46.04 | 85.71% |
| LN2 | 6.60 | 93.56 | 45.91 | 83.36% |
| LN3 | 7.60 | 93.71 | 48.00 | 75.61% |
| YN1 | 7.51 | 93.34 | 40.11 | 84.52% |
| YN2 | 6.59 | 93.35 | 43.87 | 80.76% |
| YN3 | 5.83 | 93.69 | 42.24 | 80.83% |
| SX1 | 5.89 | 93.21 | 43.86 | 85.54% |
| SX2 | 6.53 | 94.01 | 43.43 | 86.42% |
| SX3 | 8.89 | 93.86 | 47.47 | 82.07% |
| NMG1 | 7.67 | 93.72 | 44.78 | 85.68% |
| NMG2 | 6.62 | 93.55 | 44.10 | 84.62% |
| NMG3 | 5.79 | 93.67 | 46.62 | 75.24% |
| ONMG1 | 6.99 | 92.96 | 45.38 | 83.60% |
| ONMG2 | 8.16 | 93.51 | 42.56 | 86.57% |
| ONMG3 | 6.43 | 93.61 | 46.65 | 78.80% |

Supplementary Tab.2 Evaluation of the three-dimensional protein structure prediction model.

| Model | Clashscore^a^ | Favored rotamers | Ramachandran favored | MolProbity score^b^ |
| --- | --- | --- | --- | --- |
| GST | 1.43 | 96.28% | 98.16% | 0.88 |
| GCLC | 0.95 | 96.30% | 93.83% | 1.20 |
| IDH1 | 0.61 | 98.01% | 95.81% | 0.99 |
| IDH2 | 0.15 | 98.38% | 95.35% | 0.88 |
| icd | 0.34 | 98.39% | 95.89% | 0.91 |
| GLUD1_2 | 0.71 | 96.89% | 96.51% | 0.96 |
| gdhA | 0 | 100.00% | 96.05% | 0.77 |
| GOT1 | 1.24 | 97.98% | 98.28% | 0.84 |

^a^Clashscore is the number of serious steric overlaps (>0.4 Å) per 1000 atoms.

^b^MolProbity score combines the clashscore, rotamer, and Ramachandran evaluations into a single score, normalized to be on the same scale as X-ray resolution.


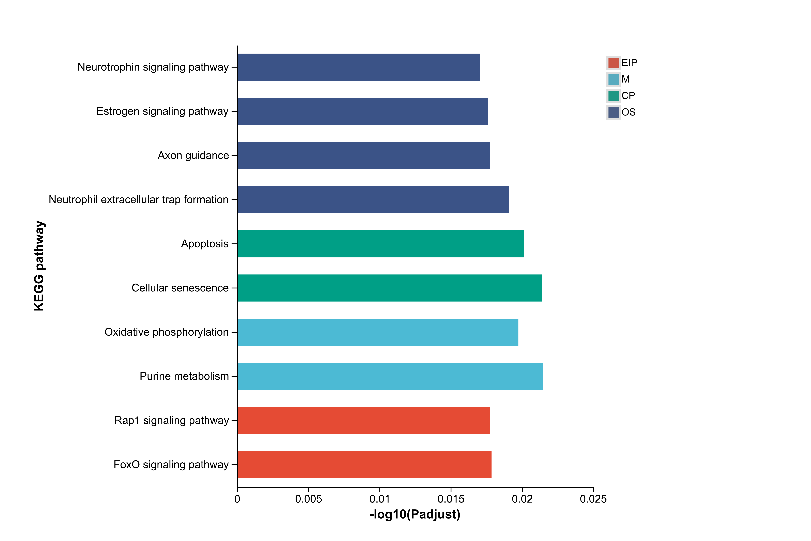

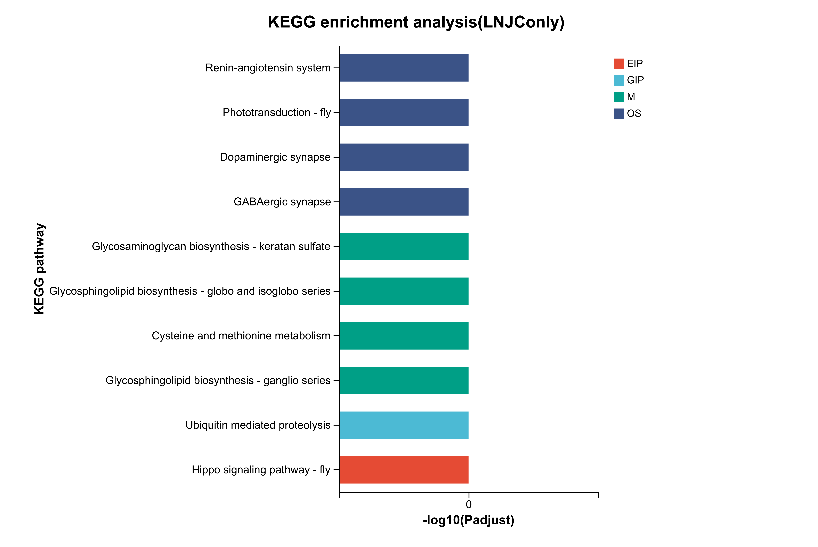


Supplementary Fig.1 Enrichment analysis of KEGG genes with specific differential expression in LN population during the initiation process of diapause (left) and the termination process of diapause (right)


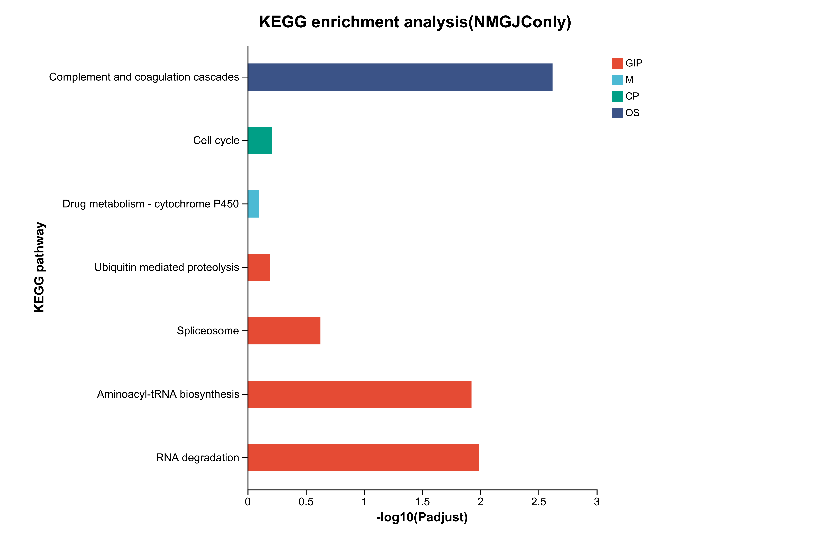

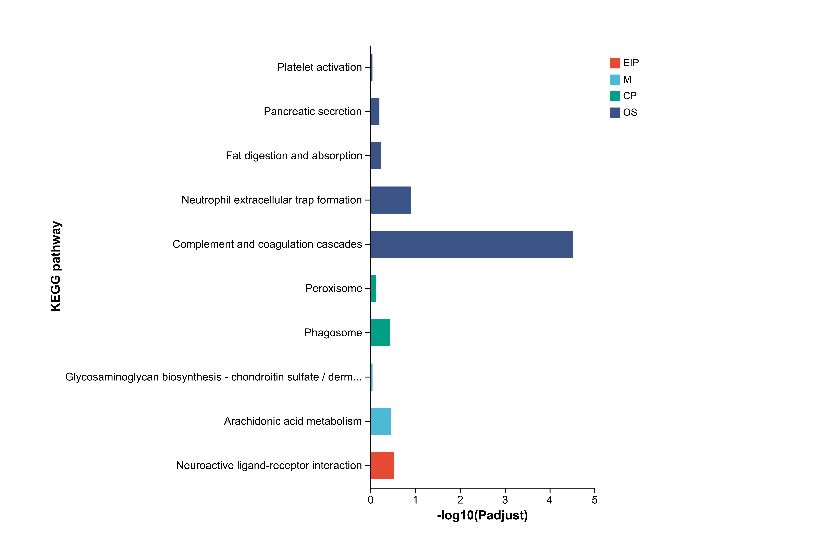


Supplementary Fig.2 Enrichment analysis of KEGG genes with specific differential expression in NMG population during the initiation process of diapause (left) and the termination process of diapause (right)


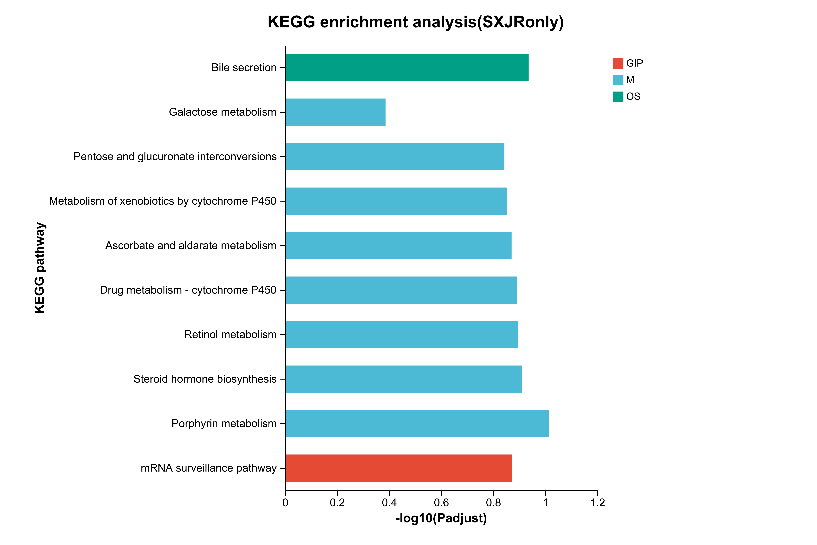

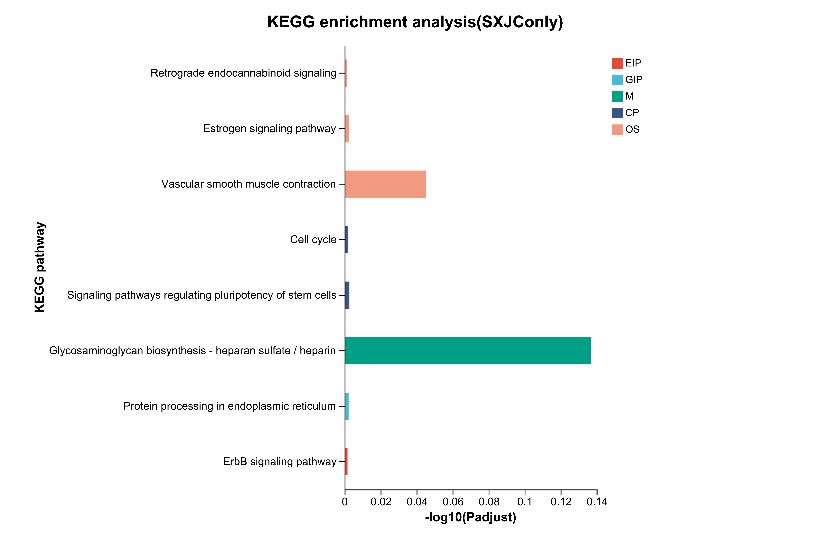


Supplementary Fig.3 Enrichment analysis of KEGG genes with specific differential expression in SX population during the initiation process of diapause (left) and the termination process of diapause (right)


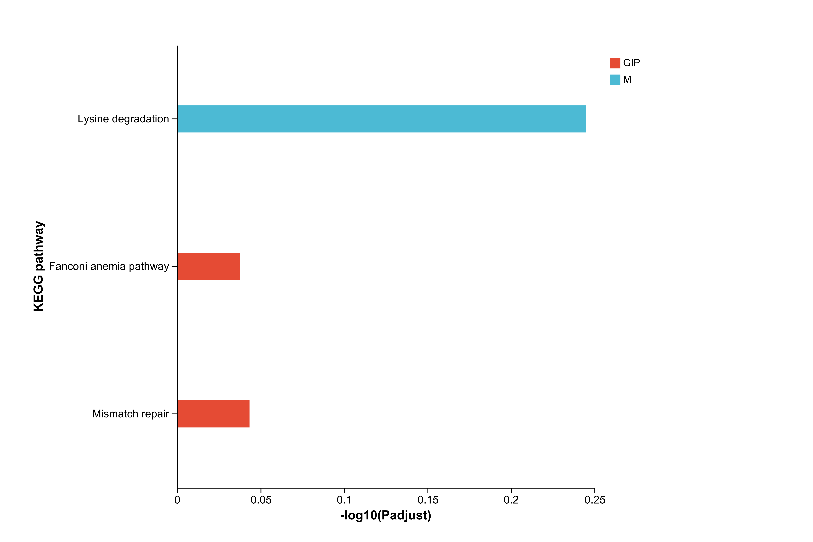

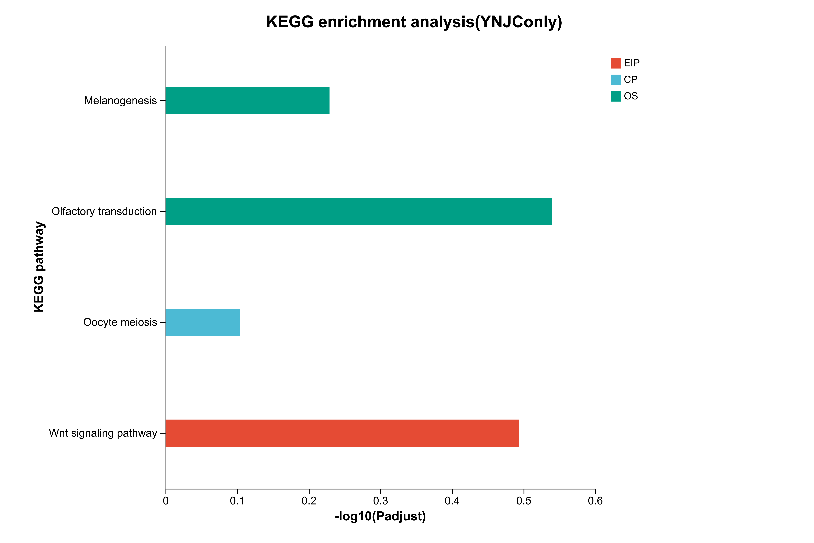


Supplementary Fig.4 Enrichment analysis of KEGG genes with specific differential expression in YN population during the initiation process of diapause (left) and the termination process of diapause (right)


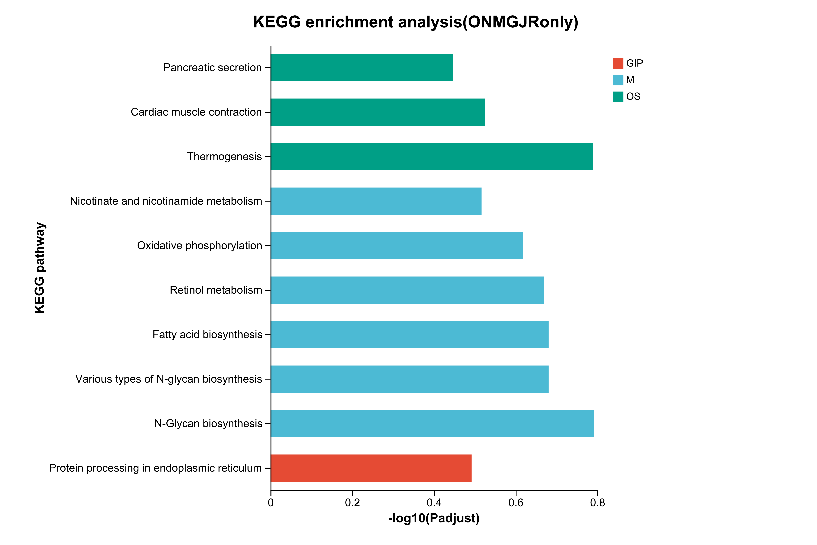

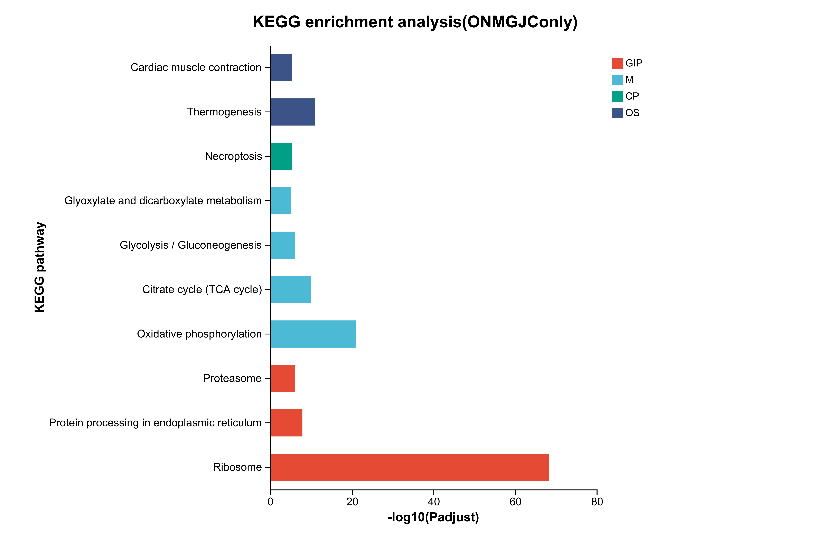


Supplementary Fig.5 Enrichment analysis of KEGG genes with specific differential expression in ONMG population during the initiation process of diapause (left) and the termination process of diapause (right)


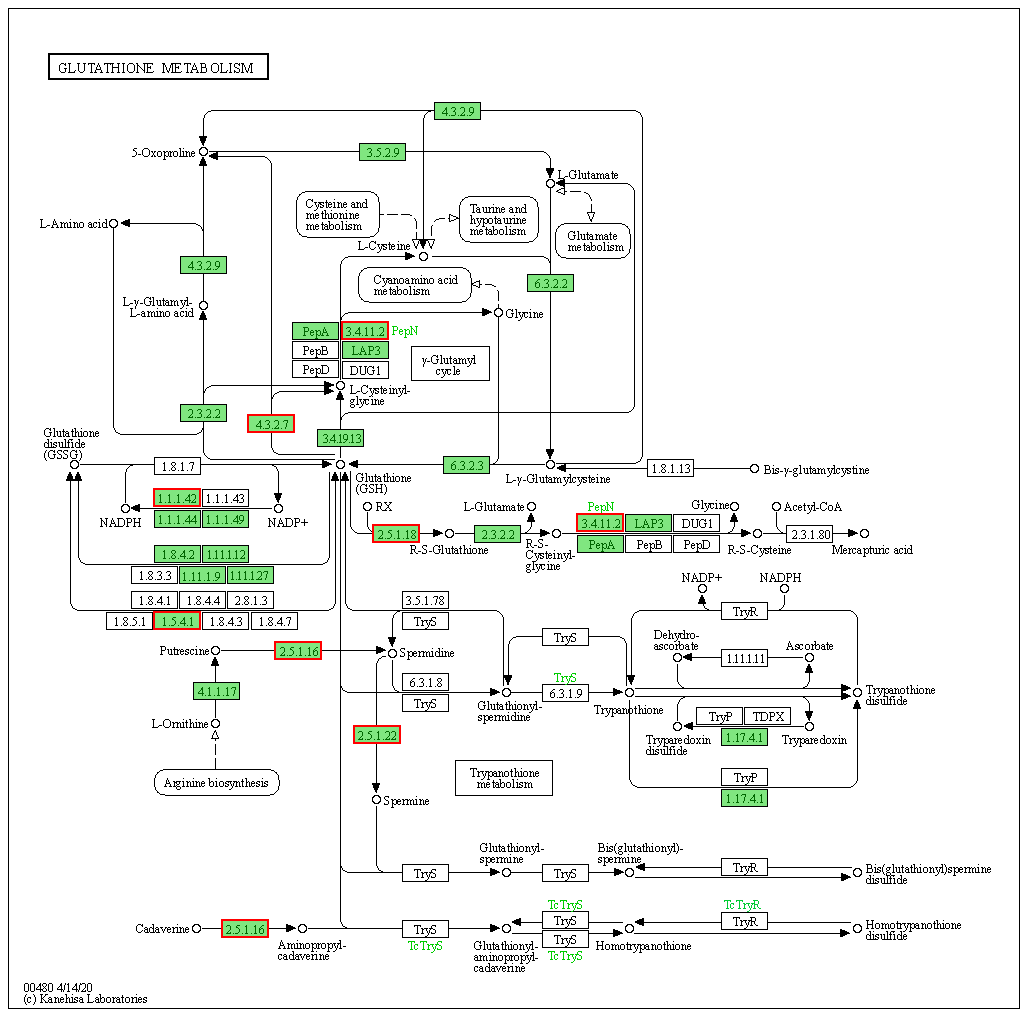


Supplementary Fig.6 Glutathione metaboLic pathway during the initiation process of diapause


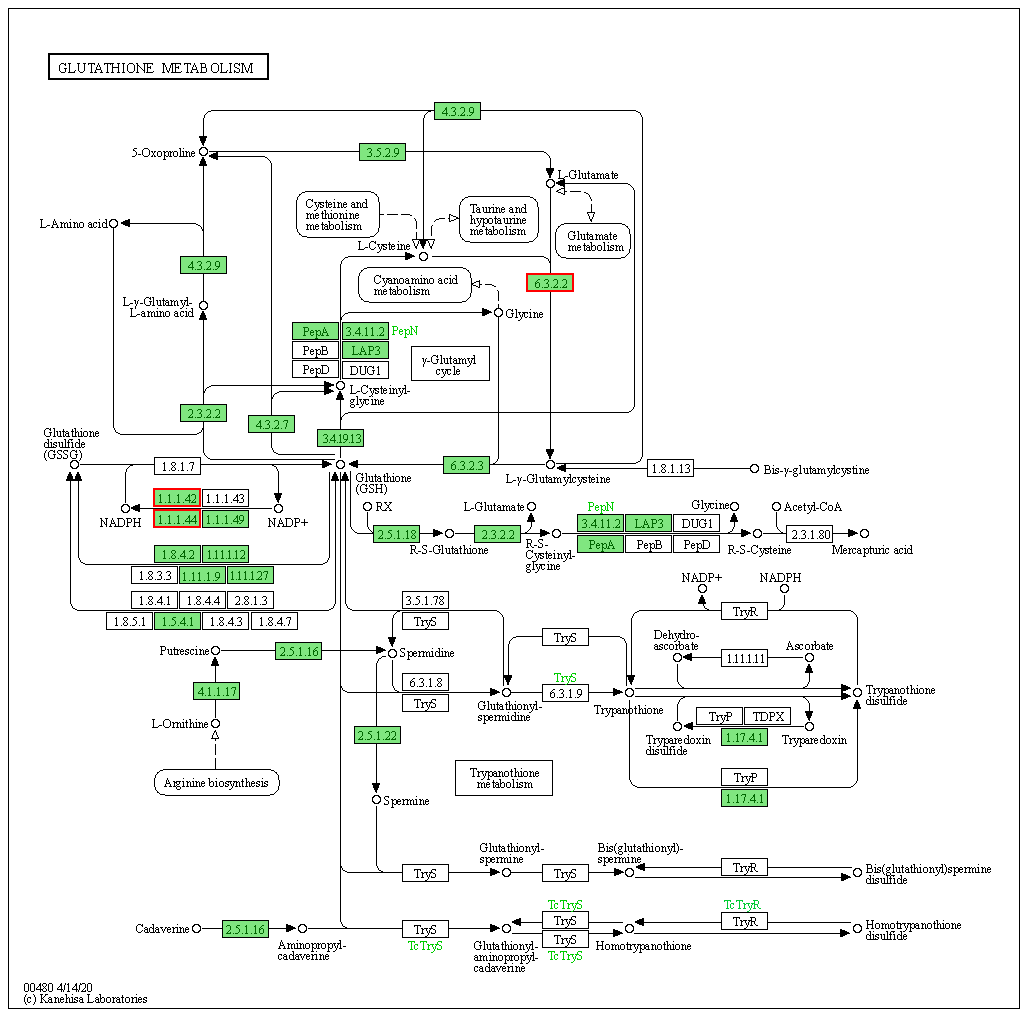


Supplementary Fig.7 Glutathione metaboLic pathway diagram during the termination process of diapause


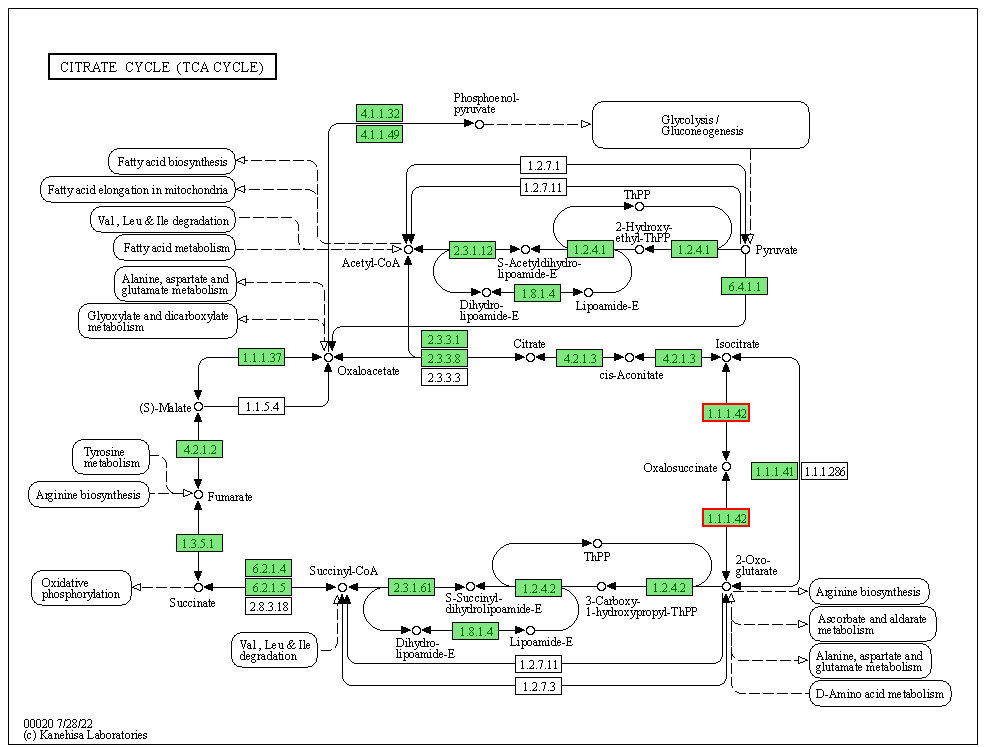


Supplementary Fig.8 The pathway map of citric acid metaboLism during the initiation process of diapause


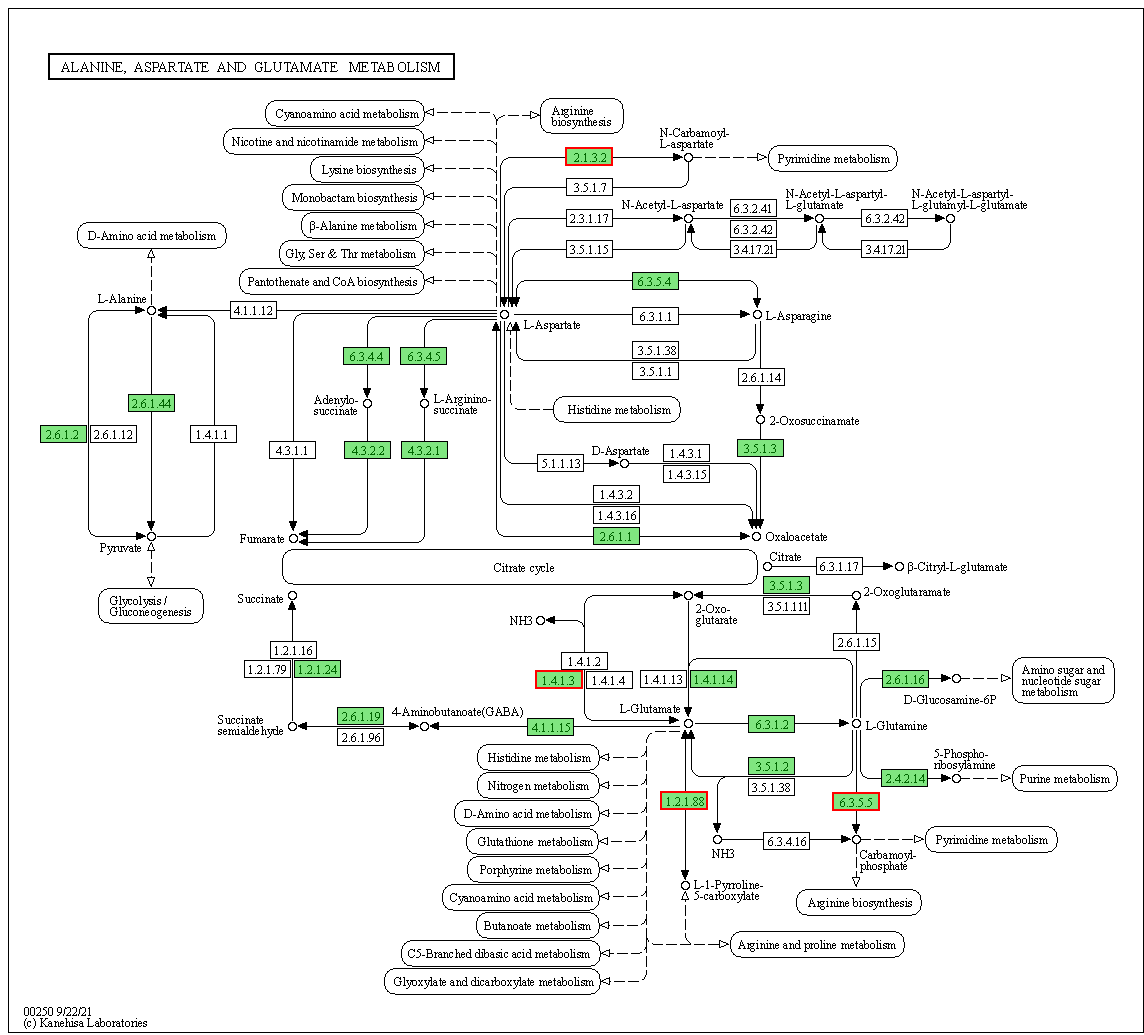


Supplementary Fig.9 Alanine, aspartic acid and glutamic acid metaboLic pathways during the initiation process of diapause


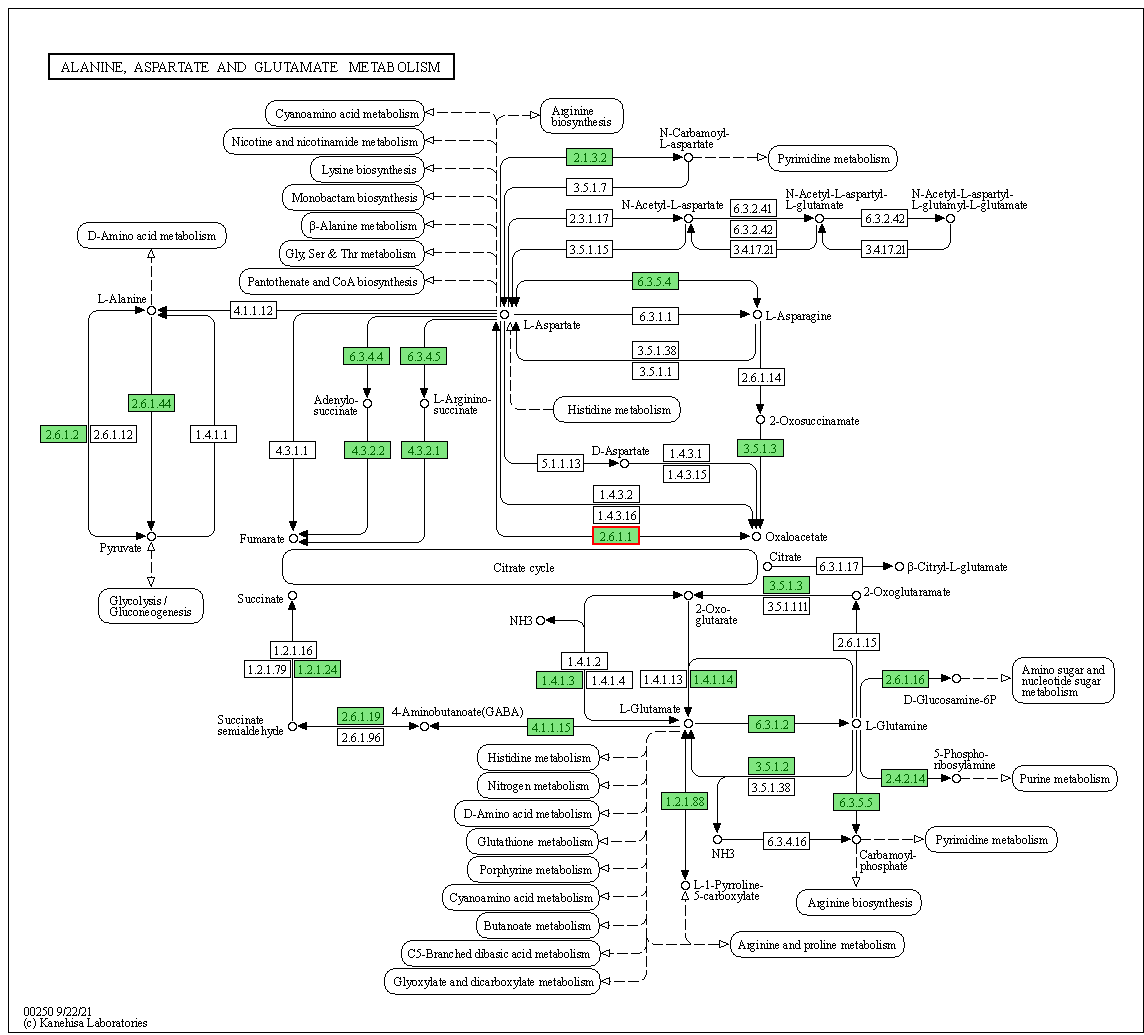


Supplementary Fig.10 Alanine, aspartic acid and glutamic acid metaboLic pathways during the termination process of diapause


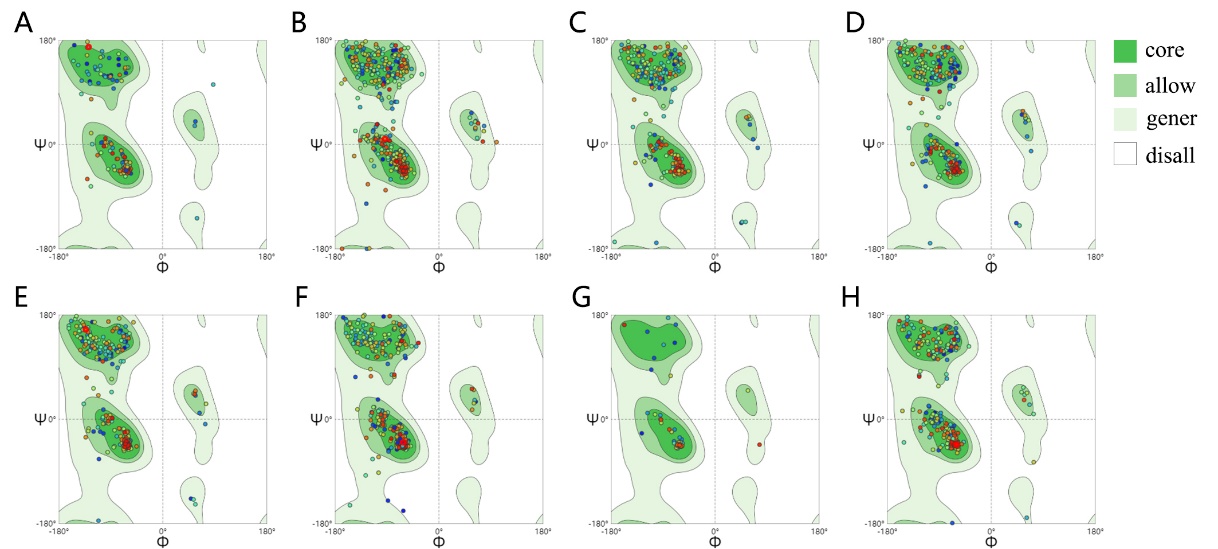


Supplementary Fig.11 Ramachandran plots verification of three-dimensional protein structure prediction. GST (A), GCLC (B), IDH1 (C), IDH2 (D), icd (E), GLUD1_2 (F), gdhA (G), GOT1 (H).
