# Supplementary material for: Identification of differentially expressed genes and proteins related to diapause in Lymantria dispar: Insights for the mechanism of diapause from transcriptome and proteome analyses
Source: PLoS One. 2025 Jun 25;20(6):e0316065. doi: 10.1371/journal.pone.0316065 (PMC12192293; doi:10.1371/journal.pone.0316065)
Supplement: Attachment — Submitted filename: Response to reviewers.docx [file pone.0316065.s023.docx]

**Response to Reviewer**

**Comment 1:**

***The writing is imprecise and confusing, with numerous inappropriate statements that hinder the effective communication of the message. So i think the authors should reorganize the content and necessitate substantial revisions to the manuscript***

**Answer**:

We sincerely thank the reviewer for their attention to the writing quality of our manuscript. To ensure the scientific accuracy and clarity of our study, we have made substantial revisions throughout the entire manuscript. These include language refinement, structural reorganization, clarification of key concepts, and optimization of logical coherence. The specific modifications are summarized as follows:

**1. Introduction**

We made extensive and in-depth revisions to the Introduction section, rewriting the majority of its content to improve clarity and enhance logical flow. Key improvements include:

(1) **Structural Reorganization:** In the original version, information about the ecological background of *Lymantria dispar*, its damage, and previous research on diapause was scattered and lacked cohesion. We have now restructured the Introduction to follow a clear logical sequence: background - current knowledge - knowledge gaps - research objectives, effectively conveying the research rationale and importance.

(2) **Concept Clarification:** Key biological terms such as the classification of L. dispar and the types of diapause have been revised for greater precision. To improve conceptual continuity, the explanation of diapause types now precedes the discussion of its occurrence in L. dispar.

(3) **Language Refinement:** We have streamlined redundant or repetitive descriptions. For instance, previously separate paragraphs on environmental factors influencing diapause have now been merged into a coherent section. Each paragraph is now centered around a single topic and begins with a topic sentence to strengthen inter-paragraph logic.

(4) **Stylistic Improvement:** We have refined wording for greater professionalism and fluency. Ambiguous or repetitive terms were replaced with more accurate expressions (e.g., “numerous outbreaks” → “extensive outbreaks”).

**2. Materials and Methods**

We clarified previously vague content, such as the exact definition of the pre-diapause, diapause, and post-diapause stages. Specifically, we added the number of days and temperature conditions corresponding to each stage (Lines 118–121). Furthermore, we replaced inconsistent descriptions of developmental transitions with precise terms: diapause initiation process (transition from pre-diapause to diapause) and the diapause termination process (transition from diapause to post-diapause), avoiding confusion present in the original version (Lines 329–333).

**3. Results**

The Results section was substantially revised, with many additions, deletions, and reorganization. Major improvements include:

(1) **Population-specific analysis:** We added new analyses of differentially expressed genes (DEGs) and pathways across five geographically distinct populations. This highlights the combined influence of environmental and genetic factors in diapause regulation and strengthens the significance of our multi-population design.

(2) **Enhanced data transparency:** New supplementary data were added, including transcriptome quality metrics for all five populations, developmental stage-specific gene/protein expression profiles, details of 52 significantly enriched third-level KEGG pathways, and KEGG pathway maps during diapause initiation and termination.

(3) **Phylogenetic analysis revision:** We reconstructed the phylogenetic trees following standard practices and rewrote all related descriptions based on species-specific evolutionary relationships of the candidate genes.

(4) **Figure updates:** We added new figures to support KEGG enrichment and gene selection rationale, and moved tertiary structure validation figures to the supplementary materials for clarity.

**4. Discussion**

The Discussion section was completely rewritten to improve logical flow, coherence, and scientific depth. Major revisions include:

(1) **Summary of major findings:** We now provide concise discussion of the biological significance of the three main metabolic pathways (glutathione metabolism, TCA cycle, alanine, aspartate and glutamate metabolism), integrating our findings with existing literature.

(2) **Population-level insights:** We expanded the discussion to compare diapause regulatory strategies across different geographic populations, revealing adaptation mechanisms to varying environmental pressures.

(3) **Application and future outlook:** A new paragraph discusses the applied relevance, current limitations, and directions for future research.

All changes are highlighted in yellow in the revised manuscript. We also provide original and revised text excerpts with corresponding line numbers in our point-by-point response. Once again, we sincerely thank the reviewer for their constructive and insightful suggestions, which have significantly improved the clarity and quality of our manuscript.

**Comment 2:**

***In the Materials and Methods section, line 128, the authors mention that eggs during pre-diapause, diapause, and post-diapause periods were selected as samples. However, i suggest specifying the exact timing (e.g., the specific day of egg development) for greater clarity.***

**Answer:**

Thank you for this thoughtful suggestion. We have now clarified the specific timing of each diapause stage in the revised manuscript. The number of days and corresponding temperature conditions are now clearly defined in the Materials and Methods section (Lines 118–121), as follows:

*Specifically, the pre-diapause stage was defined as 0–30 days post-oviposition at 25°C, the diapause stage as 30–90 days post-oviposition at 5°C, and the post-diapause stage as 90 days post-oviposition until larval hatching (approximately 10–30 days) at 25°C.*

**Comment 3:**

***The authors conducted an analysis of the transcriptome and proteome across five species of spongy moth. What was the purpose of chose these five species? After all, there is no relevant analysis in the results section and no discussion in the***

***discussion section.***

**Answer:**

Thank you for raising this important point. In our study, we selected five geographically distinct populations of *L. dispar* (from Inner Mongolia, Shanxi, Liaoning, Yunnan, and one Asian-European hybrid population) for transcriptomic and proteomic analysis. However, in the original version, we only focused on the shared DEGs and did not elaborate on the population-specific differences in either the Results or Discussion sections.

We have now revised the manuscript to address this gap. Below are our detailed justifications and updates:

**1. Rationale for Population Selection**

Our choice of populations was based on three main scientific considerations:

(1) To investigate how diapause regulation varies across distinct environmental conditions;

(2) To assess whether genetic background influences the expression of diapause-related genes;

(3) To explore the regulatory plasticity of diapause in a hybrid population.

The four Asian-type populations span a north-to-south geographic gradient within China, representing a natural climate diversity. Meteorological data from the China Meteorological Administration (https://data.cma.cn) show the following average winter temperatures (2014–2024): Inner Mongolia: –16.2°C; Liaoning: –13.7°C; Shanxi: –5.8°C; Yunnan: 7.1°C.

These gradients likely affect the length and intensity of low-temperature exposure required to terminate diapause. By comparing gene expression patterns among these populations, we aim to identify how local adaptation may shape diapause regulation.

In addition, the inclusion of a hybrid population (Inner Mongolia male × New Jersey female) allowed us to explore the potential impact of genetic background on diapause gene regulation. Previous studies have shown that Asian-type moths typically require shorter chilling periods to terminate diapause compared to European-type moths. Although we were unable to include a pure European population due to sample limitations, the hybrid population provides valuable insights into potential maternal or biparental inheritance effects in diapause regulation.

**2. New Results Added to the Manuscript**

We have added population-specific DEG analyses during diapause initiation and termination. We identified uniquely expressed genes for each population and conducted KEGG enrichment analysis to determine the major pathways involved (Lines 356–388). These additions strengthen the multi-population comparative framework of our study.

***3.4 Population-Specific Diapause-Related Genes and Pathway Enrichment Analysis***

*KEGG enrichment analysis of population-specific DEGs during diapause initiation process and diapause termination process revealed distinct regulatory strategies among geographical populations (File S1, Supplementary Figures 1-5). Northern populations (LN, NMG) preferentially activated stress response pathways during diapause initiation process: the LN population regulated neuroendocrine plasticity through the Neurotrophin signaling pathway, while the NMG population enhanced immune tolerance via the Complement and coagulation cascades pathway, simultaneously suppressing the Cell cycle pathway to achieve developmental arrest. During diapause termination process, the LN population shifted to the Renin-angiotensin system to regulate osmotic balance, whereas the NMG population maintained immune homeostasis through sustained activity of the Complement and coagulation cascades pathway, indicating their adaptation to cold-arid environments through neuro-immune coordination. The transitional population (SX) adapted to energy demands during diapause initiation process by regulating Galactose metabolism pathway, while activating the Vascular smooth muscle contraction pathway during termination to promote fluid circulation recovery, reflecting metabolic plasticity-driven environmental adaptation. The southern population (YN) strengthened Mismatch repair during initiation process to maintain genomic stability and activated Olfactory transduction during termination process to precisely regulate hatching behavior, demonstrating refined adaptation to humid-tropical environments. The Eurasian hybrid population (ONMG) integrated Oxidative phosphorylation and N-Glycan biosynthesis during diapause initiation process, and synergistically upregulated Glycolysis/Gluconeogenesis and the Citrate cycle during termination process, forming a metabolic network cascade that provided energy metabolism advantages for ecological niche expansion.*

*This hierarchical pathway divergence not only elucidates the molecular biogeography of diapause regulation but also reveals evolutionary trade-offs in key biological functional modules: northern populations optimized survival costs through signal transduction, transitional and southern populations ensured genetic adaptation via genomic stability, and hybrid populations enhanced niche adaptability through metabolic network expansion.*

**3. Expanded Discussion on Population-Specific Regulation**

We now include a discussion of the biological significance of population-specific gene expression patterns (Lines 526–536). The analysis shows that northern populations (e.g., Inner Mongolia and Liaoning) exhibit enhanced hormonal and metabolic regulation to cope with cold stress, while southern populations (e.g., Yunnan) rely more on antioxidant and cellular protective mechanisms. The hybrid population exhibited mixed regulatory features, suggesting high plasticity in gene regulation. These findings highlight the dual role of environmental and genetic factors in shaping diapause strategies and contribute to a better understanding of geographic adaptation in L. dispar.

1.	Gray, D.R., et al., Toward a Model of Gypsy Moth Egg Phenology: Using Respiration Rates of Individual Eggs to Determine Temperature–Time Requirements of Prediapause Development. 1991(6): p. 1645-1652.

2.	Bell, R.A., Manipulation of diapause in the gypsy moth, *Lymantria dispar* L., by application of KK-42 and precocious chilling of eggs. Journal of Insect Physiology. 1996. 42(6): p. 557-563.

3.	Tauber, M.J., et al., Dormancy in *Lymantria dispar* (Lepidoptera: Lymantriidae): Analysis of Photoperiodic and Thermal Responses. 1990(3): p. 494-503.

4.	Wei, J., et al., Impact of temperature on postdiapause and diapause of the Asian gypsy moth, *Lymantria dispar asiatica*. Journal of Insect Science, 2014. 14: 5.

**Comment 4:**

***According to the authors description, transcriptomic analyses were conducted on five species. However, only one dataset (including Q30, mapping rates, etc.) is presented in the results. Why? It is advisable to include detailed data in the supplementary materials or upload them to the NCBI database for comprehensive accessibility.***

**Answer:**

We appreciate the reviewer’s concern regarding the completeness of our data and fully understand the importance of providing comprehensive datasets to ensure transparency and reproducibility in scientific research. In the original submission, we indeed performed transcriptome sequencing for five populations of *L. dispar*, but only included the lowest quality metrics to briefly reflect the overall sequencing quality in the main text. This decision was originally made to maintain conciseness. However, we acknowledge that this approach may have led to insufficient detail. Therefore, in this revised version, we have made the following changes to improve data accessibility and transparency in response to the reviewer’s suggestions:

**1. Addition of Supplementary Materials with Full Quality Metrics for All Five Populations**

We have compiled the complete transcriptome sequencing quality statistics for all five populations into a table, which is now included as Supplementary Table 1 (File S1). This table provides details such as the number of clean reads, Q30 values, GC content, and mapped ratio for each population, offering a clear view of the sequencing quality across all samples.

**2. Upload of Raw Sequencing Data to NCBI SRA with Access Information**

Following the reviewer’s recommendation, we have submitted all raw transcriptome sequencing data for the five populations to the NCBI Sequence Read Archive (SRA). The corresponding SRA accession number (**BioProject ID: PRJNA1238095**) is now provided in the “Data Availability” section of the manuscript. The uploaded dataset includes clean FASTQ files, experimental design details, and sample metadata.

**Comment 5:**

***In the results section, the authors described "The period from pre-diapause to mid-diapause was termed as the diapause initiation stage, whereas the period from mid-diapause to post-diapause was termed as the diapause termination stage." Which period constitutes the "diapause maintenance stage."? Additionally, the Materials and Methods section indicates that diapause egg samples were selected, yet the results lack data pertaining to the diapause stage.***

**Answer:**

Thank you for your insightful comment. We address your concerns in two parts:

**1. Clarification and Revision of Diapause Stage Definitions**

We acknowledge that the original phrasing may have led to ambiguity. In *L. dispar*, diapause consists of three well-defined phases:

**Pre-diapause**: Also referred to as the preparation phase, during which embryonic development is completed in preparation for diapause.

**Diapause (mid-diapause)**: This is the maintenance stage, characterized by developmental arrest and low metabolic activity.

**Post-diapause**: Also known as the termination phase, when development resumes.

We refer to the transition from pre- to mid-diapause as the **diapause initiation process**, and the transition from mid- to post-diapause as the **diapause termination process**. This terminology is now clearly explained in the manuscript (Lines 329–333):

*By comparing differentially expressed genes and proteins among the three stages (pre-diapause, diapause, and post-diapause), we screened differentially expressed genes and proteins involved in the diapause initiation process (transition from pre-diapause to diapause) and the diapause termination process (transition from diapause to post-diapause).*

**2. Addition of Diapause Phase Data**

We also appreciate your attention to data completeness. In the original manuscript, we focused only on differentially expressed genes between stages and omitted individual-stage expression profiles. In this revision, we have added Supplementary Files 14 and 15, which contain the expression profiles of all assembled genes and proteins at pre-diapause, diapause, and post-diapause stages for each of the five populations. We believe this significantly improves the clarity and thoroughness of our dataset.

**Comment 6:**

***In the results, lines 341-351, the authors said "470 showing an upregulation and 465 showing a downregulation... 822 being upregulated and 264 being downregulated...". It is necessary to clarify the reference group used for these upregulated and downregulated comparisons.***

**Answer:**

Thank you for identifying this important point. We have now clarified the reference groups used in these comparisons:

For genes described as upregulated or downregulated during diapause initiation process, the pre-diapause group was used as the reference when compared with the diapause group.

For changes during diapause termination process, the diapause group served as the reference when compared with the post-diapause group.

These clarifications have been added to the revised manuscript (Lines 334–348).

**Comment 7:**

***In line 367, the full name of "CP" should be provided.***

**Answer:**

Thank you for your careful reading. We have now corrected this oversight by defining CP (Cellular Processes) where it first appears in the text (Line 320).

**Comment 8:**

***In the results section titled "3.4 Selection of Diapause-Associated Genes in Spongy Moths", it should added detailed data, such as figures, to substantiate the description.***

**Answer:**

We appreciate the reviewer’s recommendation. In response, we have made the following enhancements:

Added a new Figure 6 to illustrate the KEGG pathway enrichment results for the shared differentially expressed genes across diapause stages in five populations.

Supplementary Files 20 and 21 now contain detailed information on all significantly enriched KEGG pathways (P < 0.05) identified during diapause initiation process and termination process.

Supplementary Figures 6–S10 (File S1) visualize the structure and enrichment of three key pathways: the glutathione metabolism pathway, the citrate cycle (TCA) pathway, and the alanine, aspartate, and glutamate metabolism pathway.

These additions provide a more comprehensive and transparent presentation of our gene selection process.

**Comment 9:**

***In the results section titled "3.4 Selection of Diapause-Associated Genes in Spongy Moths", why authors focus and selected these genes (GST, GCLC, IDH1, etc.) for in-depth analysis? In deed, there are many differentially expressed genes. Is this selection based on subjective choice or prior analyses? Moreover, i recommended that the authors should integrate transcriptomic and proteomic analyses to explore the diapause related genes.***

**Answer:**

Thank you very much for your insightful comment. In our study, we performed KEGG enrichment analysis on the DEGs shared across five populations during both the diapause initiation and termination processes. This yielded 52 significantly enriched level-3 pathways. From these, we focused on three pathways that showed the strongest biological relevance to diapause regulation: glutathione metabolism, the citrate cycle (TCA cycle), and alanine, aspartate, and glutamate metabolism. We explain the rationale for this selection below, along with justification for excluding the other 49 pathways, and how functional validation supports the biological importance of these genes.

**1. Rationale for Selecting the Three Key Pathways and Associated Genes**

（1）**Glutathione Metabolism Pathway**: This pathway is widely recognized for maintaining redox homeostasis and defending against oxidative stress during diapause [1–3]. Key genes like GST and GCLC play critical roles: GST is involved in detoxification, while GCLC is responsible for glutathione synthesis, thus regulating the antioxidant capacity of cells. Since oxidative stress is a hallmark of diapause, the activity of this pathway is essential for diapause maintenance. Prior studies have also shown significant expression changes in *GST* [4,5] and *GCLC* [6,7] during insect diapause, justifying their prioritization.

（2）**Citrate Cycle**: As the core of cellular energy metabolism, the TCA cycle is known to be suppressed during diapause [8,9]. Only three DEGs across all populations were enriched in this pathway: *IDH1*, *IDH2*, and *icd*, which catalyze the conversion of isocitrate to α-ketoglutarate, producing NADPH and playing a dual role in oxidative stress resistance and energy regulation.

（3）**Alanine, Aspartate, and Glutamate Metabolism Pathway**: This pathway connects amino acid metabolism with energy metabolism, crucial for maintaining metabolic balance during diapause [10,11]. Among four enriched genes, we excluded *CAD* (involved in nucleotide metabolism) and selected *GLUD1_2*, *gdhA*, and *GOT1*, which bridge amino acid metabolism and the TCA cycle, regulating nitrogen balance and energy supply under low metabolic conditions typical of diapause.

**2. Reasons for Excluding the Remaining 49 Pathways**

To ensure biological relevance of screened genes, the remaining 49 enriched pathways were systematically evaluated, with the rationale for their exclusion detailed as follows:

| **Pathway** | **Exclusion Rationale** |
| --- | --- |
| Metabolism | |
| Phenylalanine, tyrosine and tryptophan biosynthesis | Primarily involved in aromatic amino acid synthesis, not categorized as a core metabolic regulatory pathway during diapause.\ |
| Phenylalanine metabolism | Mainly mediates neurotransmitter synthesis and degradation of metabolic byproducts, exhibiting weak regulatory roles in diapause. |
| Tyrosine metabolism | Primarily participates in melanin and catecholamine synthesis. |
| Arginine biosynthesis | Primarily associated with urea cycle and protein metabolism, showing limited direct relevance to diapause regulation. |
| Arginine and proline metabolism | Broad functional scope, not identified as a core regulatory mechanism for diapause. |
| Pentose phosphate pathway | Primarily supports nucleotide synthesis and NADPH production, lacking specificity in diapause regulation. |
| Glycerolipid metabolism | Affects energy reserves, but core energy regulation in diapause relies predominantly on TCA cycle and amino acid metabolism. |
| Purine metabolism | Primarily impacts nucleotide synthesis, with minimal direct effects on diapause. |
| Oxidative phosphorylation | Dependent on TCA cycle, but changes in oxidative phosphorylation may merely reflect downstream consequences of TCA cycle adjustments. |
| Terpenoid backbone biosynthesis | Terpenoids in insects primarily synthesize juvenile hormone (JH) and ecdysone, not directly governing diapause mechanisms. |
| Biosynthesis of unsaturated fatty acids | Fatty acids serve as energy stores during diapause, but regulation focuses on metabolic control rather than biosynthesis. |
| Genetic Information Processing | |
| Protein processing in endoplasmic reticulum | Activated during diapause for protein folding and stress responses, but not part of core metabolic regulation. |
| Proteasome | Primarily regulates protein degradation, showing lower priority compared to metabolic regulation in diapause. |
| mRNA surveillance pathway | Mainly influences gene expression regulation, not constituting a core diapause mechanism. |
| Nucleocytoplasmic transport |  |
| Spliceosome |  |
| Cell adhesion molecules | Primarily affects cell-cell interactions, with no direct link to core diapause regulation. |
| Environmental Information Processing | |
| Notch signaling pathway | These pathways primarily regulate insect development and immune responses, functioning in developmental adaptation rather than diapause-specific control. |
| Wnt signaling pathway |  |
| MAPK signaling pathway - fly |  |
| cAMP signaling pathway |  |
| Ras signaling pathway |  |
| AMPK signaling pathway |  |
| Phospholipase D signaling pathway | Mainly regulates transmembrane signal transduction. |
| Neuroactive ligand-receptor interaction | Governs nervous system activity, whereas diapause regulation is not primarily driven by neural signaling. |
| Organismal Systems | |
| Toll and Imd signaling pathway | These pathways primarily regulate insect development and immune responses, functioning in developmental adaptation rather than diapause-specific control. |
| NOD-like receptor signaling pathway |  |
| Fc gamma R-mediated phagocytosis | Focuses on immune system functions, whereas diapause regulation centers on metabolic control. |
| Neutrophil extracellular trap formation |  |
| Oxytocin signaling pathway | Primarily vertebrate-specific endocrine pathways, with limited relevance to insect diapause. |
| Pancreatic secretion |  |
| Renin secretion |  |
| Aldosterone synthesis and secretion |  |
| Vascular smooth muscle contraction |  |
| PPAR signaling pathway | Broadly regulates lipid metabolism and energy homeostasis, but TCA cycle and amino acid metabolism provide more direct regulatory nodes. |
| Longevity regulating pathway - multiple species | Diapause represents short-term metabolic suppression rather than prolonged lifespan mechanisms. |
| Longevity regulating pathway - worm |  |
| Thermogenesis | Insect diapause employs metabolic suppression rather than active thermogenesis, rendering this pathway functionally limited. |
| Antigen processing and presentation | Primarily mediates immune system functions. |
| Protein digestion and absorption | Mainly regulates nutrient absorption, unrelated to diapause regulation. |
| Cellular Processes | |
| Autophagy - animal | Regulates cellular degradation processes, weakly connected to diapause-associated metabolic control. |
| Lysosome |  |
| Peroxisome |  |
| Apoptosis | Diapause involves metabolic suppression rather than programmed cell death mechanisms. |
| Ferroptosis |  |
| Apoptosis - fly |  |
| Endocytosis | Primarily mediates substance uptake and signaling, not part of core diapause metabolism. |
| Tight junction | Governs intercellular barrier functions without direct roles in insect diapause. |
| Regulation of actin cytoskeleton | Mainly involved in cell motility and morphological changes, demonstrating weak diapause relevance. |

**3. Functional Validation of Selected Genes**

We conducted RNAi experiments targeting the selected genes (*GCLC*, *IDH1*, *IDH2*, and *GLUD1_2*) to verify their functional roles. The results confirmed that silencing these genes significantly altered diapause hormone levels and reduced hatching rates, providing strong experimental evidence supporting their regulatory role in diapause.

Our selection was not subjective, but based on multi-omics evidence, pathway-level enrichment, biological relevance, and experimental validation. This systematic approach ensured scientific rigor in identifying core genes regulating diapause in *L. dispar*.

1.	Zhang, M.S., et al., Metabolic and transcriptional regulation of reproductive diapause in *Arma* *chinensis*. Iscience, 2025. 28(3).

2.	Moreira, D.C., et al., Changes in metabolism and antioxidant systems during tropical diapause in the sunflower caterpillar *Chlosyne lacinia* (Lepidoptera: Nymphalidae). Insect Biochemistry and Molecular Biology, 2021. 134.

3.	Zhao, L.C., et al., Changes in glutathione redox cycle during diapause determination and termination in the bivoltine silkworm, *Bombyx mori*. Insect Science, 2014. 21(1): p. 39-46.

4.	Santos, P.K.F., et al., Diapause in a tropical oil-collecting bee: molecular basis unveiled by RNA-Seq. Bmc Genomics, 2018. 19.

5.	Bao, B. and W.H. Xu, Identification of gene expression changes associated with the initiation of diapause in the brain of the cotton bollworm, *Helicoverpa armigera*. Bmc Genomics, 2011. 12.

6.	Robbins, H.M., et al., Diapause termination and development of encysted *Artemia* embryos: roles for nitric oxide and hydrogen peroxide. Journal of Experimental Biology, 2010. 213(9): p. 1464-1470.

7.	Jovanovic-Galovic, A., et al., Antioxidant Defense in mitochondria during diapause and postdiapause development of European corn borer (*Ostrinia nubilalis*, Hubn.). Archives of Insect Biochemistry and Physiology, 2007. 64(3): p. 111-119.

8.	Ren, X.Y., et al., Proteomic research on diapause-related proteins in the female ladybird, *Coccinella septempunctata* L. Bulletin of Entomological Research, 2016. 106(2): p. 168-174.

9.	Michaud, M.R. and D.L. Denlinger, Shifts in the carbohydrate, polyol, and amino acid pools during rapid cold-hardening and diapause-associated cold-hardening in flesh flies (*Sarcophaga crassipalpis*): a metabolomic comparison. Journal of Comparative Physiology B-Biochemical Systems and Environmental Physiology, 2007. 177(7): p. 753-763.

10.	Zhang, Q., et al., Proteomic and metabolomic profiles of larval hemolymph associated with diapause in the cotton bollworm, *Helicoverpa armigera*. Bmc Genomics, 2013. 14.

11.	Ragland, G.J., et al., Developmental trajectories of gene expression reveal candidates for diapause termination: a key life-history transition in the apple maggot fly *Rhagoletis pomonella*. Journal of Experimental Biology, 2011. 214(23): p. 3948-3959.

**Comment 10:**

***In the section titled "3.5 Diapause-Associated Gene Homology and Phylogenetic Analysis", the evolutionary tree results are improperly described. Why the authors described the evolutionary relationships of the genes in different families? The authors should describe the evolutionary relationship of each gene across species. Furthermore, the description beginning with "The amino acid...various closely related species" has already been detailed in the Materials and Methods section and should be removed for redundancy.***

**Answer:**

We sincerely appreciate the valuable suggestions for improving the manuscript's clarity and scientific rigor. In response to the inadvertent conflation of evolutionary relationships across different gene families in the original discussion, we have systematically reanalyzed the evolutionary relationships of each diapause-associated gene. Separate phylogenetic trees were reconstructed for distinct gene families (Figure 7), with new evolutionary tree analyses incorporated for each gene. Redundant content duplicating "Materials and Methods" descriptions has been removed from the Results section. The revised content (Lines 437-458) now presents updated phylogenetic analyses and corresponding evolutionary interpretations.

*This study investigated the evolutionary relationships of eight diapause-related genes in L. dispar through phylogenetic analysis (Fig 7).*

*(1) The phylogenetic analysis of LdGST (Fig 7A) revealed that this gene formed a distinct clade with GST genes from Noctuidae species, supporting its conserved evolutionary pattern within Lepidoptera.*

*(2) LdGCLC (Fig 7B) clustered with the GCLC gene of T. ni and further grouped with H. zea GCLC to form an evolutionary branch, indicating high conservation among Lepidoptera. Significant evolutionary divergence was observed between LdGCLC and homologs from Nematocida displodere and Tribolium castaneum.*

*(3) Phylogenetic analysis of LdGLUD1_2 and LdgdhA (Fig 7C) demonstrated that LdGLUD1_2 formed an independent clade with GLUD1_2 genes from Ctenocephalides felis and Caenorhabditis elegans. LdgdhA exhibited the closest evolutionary relationship with the gdhA gene of Amyelois transitella, suggesting functional conservation in Lepidoptera.*

*(4) The phylogenetic reconstruction of LdIDH1, LdIDH2, and Ldicd (Fig 7D) showed that both LdIDH1 and LdIDH2 clustered within the Lepidoptera branch, forming a group with IDH genes from B. mori, H. armigera, and S. litura. In contrast, Ldicd grouped with the icd gene of Trichogramma pretiosum.*

*(5) Analysis of LdGOT1 (Fig 7E) revealed that this gene formed an independent evolutionary branch with Cordylochernes scorpioides GOT1, which subsequently clustered as a sister group to an evolutionary cluster containing H. zea, S. frugiperda, and T. ni.*

**Comment 11:**

***In the section of n3.6 Prediction of Three-Dimensional Structures of Diapause-Associated Proteins in Spongy Moths", Figure 8 and Table 3 pertain to the reliability verification of the tertiary structure model. These can be moved to the supplementary materials.***

**Answer:**

We appreciate the reviewers' suggestions regarding the presentation of three-dimensional structure prediction results and concur that relocating model reliability validation data to supplementary materials enhances focus on core findings. The original Figure 8 and Table 3, which primarily served to verify model quality, have been transferred to Supplementary File 1 (Figure S11 and Table S2). The main text has been appropriately streamlined to eliminate redundant validation details while preserving critical validation metrics to ensure readers can still assess model credibility. The revised manuscript section (Lines 473-477) now concisely describes structural prediction outcomes and references supplementary materials for comprehensive validation data.

*The stereochemical quality of protein three-dimensional structural models was evaluated using Ramachandran plots, with all models demonstrating >99% of amino acid residues in allowed regions (File S1, Supplementary Figure 11). Model quality was further validated through MolProbity scoring incorporating clashscore, rotamer outliers, and Ramachandran plot statistics (File S1, Supplementary Table 2).*

**Comment 12:**

***The discussion section is muddled. I would recommend authors to summarize the topic based on this research, and revising a large portion of the discussion section.***

**Answer:**

We sincerely thank the reviewers for their constructive suggestions. The Discussion section has been substantially revised to enhance logical coherence and clarity. Specifically, the original version exhibited structural deficiencies with lengthy descriptions that inadequately highlighted core findings. We have restructured this section to systematically and concisely delineate the functional roles of key metabolic pathways, emphasizing how expression changes of critical genes within these pathways specifically regulate diapause. Redundant content unrelated to the study's focus has been removed, supplemented by comprehensive discussions validating regulatory mechanisms through comparisons with existing literature.

A new subsection has been incorporated to address geographical variations in diapause regulation strategies among *L. dispar* populations. This addition compares gene expression profiles and metabolic pathway adaptations across different geographic populations under environmental pressures, thereby underscoring the significance of our multi-population experimental design (Lines 526-536).

Furthermore, we have expanded the discussion to include potential applications for pest control strategies, particularly regarding targeted intervention points identified in this study. The revised section concludes with an explicit analysis of current limitations and proposed directions for future research, providing a more balanced perspective.

Collectively, these revisions yield a more structured narrative that accentuates the study's central discoveries. Substantial improvements have been achieved in integrative analysis, organizational clarity, translational relevance, and forward-looking perspectives, with language streamlined for precision and accessibility.
